# A pilot study to determine the short-term effects of milk with differing glycaemic properties on sleep among toddlers: a randomised controlled trial

**DOI:** 10.1186/s12887-015-0393-9

**Published:** 2015-07-15

**Authors:** Snigdha Misra, Geok L Khor, Peter Mitchell, Samsul Haque, David Benton

**Affiliations:** Division of Nutrition and Dietetics, International Medical University, 57000 Kuala Lumpur, Malaysia; Department of Psychology, University of Nottingham, Malaysia Campus, 43500 Semenyih, Selangor Darul Ehsan Malaysia; Department of Psychology, Monash University, Sunway Campus, 47500 Subang Jaya, Selangor Malaysia; Department of Psychology, University of Swansea, Swansea, SA2 8PP Wales UK

**Keywords:** Glycaemic index, Toddlers, Sleep, Milk

## Abstract

**Background:**

Sleep is important for children as it directly impacts their mental and physical development. Sleep is not only influenced by the timing but also the macronutrient (carbohydrate and protein) content of meals. Glycaemic index (GI) and glycaemic load (GL) describe the quality of carbohydrates in a food and the burden of these foods on the body’s blood glucose response. Diets with a high GI/GL may increase the risk of developing obesity and type 2 diabetes mellitus in adulthood. The present study is piloted to evaluate the short-term impact of milk products with differing glycaemic properties on the sleep patterns of toddlers.

**Methods:**

Toddlers were recruited from various day care centres. Informed consent was obtained from both the mothers and the centres. A double-blind randomised controlled trial with a between-subjects design was adopted. The toddlers were randomised to either one of two types of milk with a differing GI (“Low” = 23 and “High = 65”) for a period of 3.5 days. There were no other dietary restrictions imposed except that the enrolled child did not consume any other milk during the study period. The sleep patterns were recorded using a Phillips Actiwatch-2, which was worn on the wrist for 24 h over 4 days. The parameters used to measure the sleep pattern were sleep-onset latency (SOL), total sleep time (TST), wake after sleep onset (WASO) and sleep efficiency (SE).

**Results:**

A total of 56 toddlers completed the study. The toddlers had a mean age of 19.9 ± 4.3 months. There were no significant differences (*p* > 0.05) between the two GI groups for SOL, TST, WASO and SE at the end of the feeding period.

**Conclusions:**

Sleep patterns of toddlers on low-GI milk did not differ from those with high-GI milk consumed over a short period. Future studies should consider the glycaemic effects of other foods, along with milk with differing GI, consumed for a longer feeding duration.

**Trial registration:**

ClinicalTrial.gov NCT01589003.

## Background

Sleep is important for children as it directly impacts their mental and physical development. During infancy, humans spend a majority of their time in sleep [1–4]. Sleep is recognised not only as a resting state, but also as a state of intense brain development during which neurotransmitters specific for each sleep stage impact brain maturation [2, 5, 6]. During early years of life, it is the primary activity of the brain. Sleep problems during childhood can predict emotional and behavioural problems, as well as poor cognitive function, which may persist into later childhood and adolescence [7]. Several hormones are involved in sleep and circadian rhythmicity [8]. Growth hormone levels are increased during sleep and peak immediately subsequent to sleep onset [9, 10]. Growth hormone is intermittently secreted during sleep, which could relate to the cyclic nature of slow-wave sleep [11].

Food and drinks have a major impact on sleep, and may stimulate or deter sleep. Sleep is not only influenced by the timing [7, 12] but also the macronutrient content [13, 14] of meals. A meal consumed close to bedtime is associated with sleep disturbance [7]. Macronutrients (carbohydrates and proteins) influence sleep through tryptophan (Trp). Trp serves as a precursor in the brain for serotonin. Serotonin is a neurotransmitter in the brain that acts as a sleep-inducing agent [15, 16]. Glucose activates serotonin, which in turn regulates stress adaptation and performance [16, 17]. Serotonin plays an important role in sleep, mood, appetite, temperature regulation and pain perception [15]. Carbohydrates (CHO) are found to increase the plasma concentration of Trp to that of the sum of the other large neutral amino acids (LNAAs), giving Trp a competitive advantage in gaining access into the brain [16, 17].

Glycaemic index (GI) and glycaemic load (GL) describe the quality of CHO in a food and the burden of these foods on the body’s blood glucose response. Diets with a high GI/GL may increase the risk of developing obesity and type 2 diabetes mellitus in adulthood [18, 19]. High-GI (HGI) CHO have the ability to increase the ratio of circulating Trp to LNAAs (Trp:LNAA) via a direct action of insulin, which promotes the selective muscle uptake of LNAAs [20]. Thus, a HGI meal would be expected to promote sleep via an increase in brain Trp and serotonin as the plasma Trp:LNAA increases [21].

Milk is a staple food, especially for children, as it delivers high-quality protein and essential nutrients such as calcium, riboflavin, vitamin A and zinc. Increasingly, milk is used as a delivery vehicle for other nutrients such as docosahexaenoic acid (DHA), vitamin D and iron. In Asian countries, a new category of high-value milk products for children, growing up milk (GUM), is well established. These products are used to supplement children’s diets to gain adequate nutrients. However, there is also a trend by manufacturers to increase the added sugar content of these products using a range of ingredients including sucrose, maltodextrins and corn or glucose syrups. Some of these ingredients may be useful in small amounts for increasing the palatability of the milks to encourage consumption. However, high levels of these ingredients not only dilute the nutritional value of the milk, but also increase intakes of added sugars by children. With the global trend of increasing overweight and obesity among children [22], these empty calories may be potentially harmful.

In Malaysia, children aged 1–3 years on average consumed almost 700 mL or 3.5 cups of milk a day, which exceeds the recommended 2–3 cups a day (200 mL/cup) by the Malaysian Ministry of Health (MOH) [23]. Countries around the world recommend the equivalent of 1–2 cups a day with the exception of Singapore, which recommends 750 mL or 3.5 cups a day for children ages 1–2 years [24]. Hence, there is a concern of added sugar in GUM contributing to HGI. A recent study found that some GUM in Malaysia and Indonesia with added CHO have HGI and GL scores that are up to 7 times higher than GUM with no added sugar [25]. GI and GL are linked to obesity risk and type 2 diabetes mellitus [26].

In Malaysia, the highest percentage (90.6 %) of milk drinkers was among those aged 1–3 years [24]. Malaysian children aged 1–3 years daily consumed milk that exceeded the amounts recommended by the MOH [23]. Both epidemiologic and methodological studies indicate a relationship between sleep and health [27, 28]. However, little is known about the impact of diet and nutrients on sleep [29–33]. Dietary CHO contain a large variety of sugar chains with differing metabolisms. Hence, it is not surprising that the amount of any individual CHO has no consistent influence on sleep parameters [34]. A cross-sectional study of toddlers showed that the consumption of foods with HGI was accompanied by longer sleep time (1.3 min/g), whereby the children’s diet was mostly based on family foods and sleep rhythmicity had been established [35]. The influence of energy and macronutrients on sleep clinical interventions confirms the cross-sectional observations that there is some relationship between the intake of macronutrients and sleep. However, the number of studies is small and substantial differences in study designs and methods exist. Additionally, none of the studies have examined the relationship between CHO with differing GI and the sleep characteristics among toddlers.

The purpose of this pilot study was to examine the short-term impact of low-GI (LGI) and HGI fortified milk powders on sleep characteristics among toddlers. It was hypothesised that LGI-fed toddlers would demonstrate on actigraphy a reduced total sleep time (TST), decreased sleep-onset latency (SOL), decreased waking after sleep onset (WASO) and decreased sleep efficiency (SE) as compared with the HGI group.

## Methods

### Ethics

The study protocol was approved by the Joint Ethics and Research Committee, International Medical University, Malaysia, with the Project Identification number IMU R 075/2011. The study was registered with ClinicalTrial.gov bearing identifier NCT01589003. Information about the objectives and principles of the study was given to participants. Written consent was also obtained from the parents for their toddlers to be recruited for the study.

### Study design

A double-blind randomised controlled trial (between subjects) design was adopted. The enrolled toddlers were randomised into two groups matched for age. The study milk products were commercially available in the market and had been tested for glycaemic properties (LGI and HGI). Both of these products have passed through appropriate quality assurance procedures and are safe for consumption. The test products were coded by the manufacturer in six colours with three of them corresponding to LGI and the remaining three to HGI. Each colour was assigned to one child to avoid mix up of the product packets among the toddlers at the centre. At any one time, only six toddlers (three for each product) were administered with the products. Thus, the researchers, the parents or the care takers at the day care centres were not aware of the type of test product being provided to the toddlers. The researchers carried equal numbers of the coloured test product packets to each of the centres to ensure appropriate randomisation of the milk products. A random number generator on a computer was used to randomise the toddlers.

Parents were interviewed to gather demographic and socioeconomic background characteristics of the toddlers, including the occupation of the parents. A questionnaire was used to collect information on the complementary feeding practices of the toddlers. The questions asked were as follows: type of food and beverages consumed, amount of food and the age at which the foods were introduced.

### Subjects

The study population was intended to include toddlers in the age range of 18 ± 3 months. Toddlers were included in the study if the following criteria were met: (1) age range of 18 ± 3 months, (2) free from any disease condition, (3) absence of lactose intolerance, (4) should be able to replace any other milk consumed with the test milk, and (5) toddlers with no known underlying health complications. Breast-fed toddlers were also included in the study if they were breast fed only at night. The mothers reported that the toddlers were breast fed only during the night to gradually wean from breast feeding. It was considered unethical to completely stop the toddlers from breast feeding. However, toddlers with continued breast feeding throughout the study period were excluded.

**Fig. 1:**
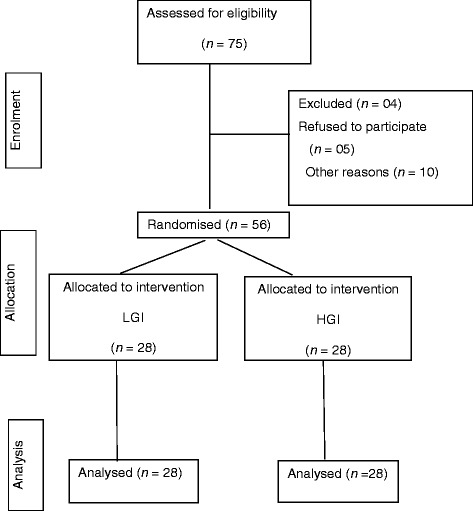
Consolidated Standards of Reporting Trials (CONSORT) diagram for the flow of enrolment of the toddlers. LGI and HGI respectively represent the groups administered a low or high glycaemic index milk product

Seventy-five parents gave a verbal consent to enrol their toddlers into the study. However, only 56 (74.6 %) parents signed up for the study. Thus, 56 toddlers were recruited for the study. Though we targeted the toddlers in the age range of 18 ± 3 months, the final age of enrolled toddlers were in the range of 14–24 months. The toddlers were recruited from different licenced day care centres in the federal territory of Kuala Lumpur and Putrajaya. The flow of the recruitment process is presented according to the Consolidated Standards of Reporting Trials (CONSORT) in Fig. 1.

### Test products

Milk with differing GI values (“Low = 23” and “High = 65”) comprised the test products. The nutrient composition of the test products is presented in Table 1. The test products are suitable for consumption by children (aged 1 year and above), and are currently available in the market. These products have been tested for glycaemic properties (low and high) in an independent accredited laboratory at the University of Sydney, Australia. Both products have passed through proper quality assurance procedures to ensure both are safe for consumption. The LGI product had a GI score of 23 and GL of 3, while the HGI product had a GI score of 65 and GL of 19. The milk formulas were isocaloric at about 29–32 kcals per scoop of 7 g of the test products. The main difference between the two products was that the HGI milk product contained added sugars contributing to a higher level of total CHO (71.7 %), while the LGI milk product had no added sugars. The total CHO of the LGI product was close to regular fresh milk (38.8 %), thereby contributing to a lower GI value.

**Table 1:** Nutrient composition of the test products

Nutrients	LGI (23)		HGI (65)	
	per 100 g	per scoop (7 g)	per 100 g	per scoop (7 g)
Energy (Kcals)	462	29	421	32
Proteins (g)	24.5	1.72	10.5	0.74
Fat (g)	22.2	1.55	11.3	0.79
CHO (g)	38.9	2.72	71.7	5.02
Vit A(mcg)	390	27.3	264	18.48
Vit D (mcg)	6.1	0.43	3.1	0.22
Calcium (mg)	830	58.1	355	24.9
Iron(mg)	11.4	0.79	5.5	0.39
Zinc (mg)	11.4	0.79	2.7	0.19

Legends: *LGI* low glycaemic index, *HGI* high glycaemic index, *CHO* carbohydrate

The toddlers were given the milk product 3 times a day at the day care centres. The composition of the administered test product was 4 scoops milk powder in 180 mL of water. The scoops were provided by the manufacturer. Each scoop of the milk product weighed 7 g. The parents were also provided with an extra packet of the same milk powder to be consumed by the toddler at home. This was to ensure that no other milk would be provided to the child beyond the day care hours. The timing of the test feed was controlled at the day care centres. However, the test feed, if provided at home, was not controlled for timing, but there was reported compliance with the serving size.

### Anthropometry

The children were weighed with minimum clothing to the nearest 10 g on a mechanical 2–16 kg capacity baby scale (Seca). The recumbent length (of children up to 2 years) or the standing height (of children 2 years and above) was measured to the nearest mm. The measurements were standardised according to the WHO recommended method [36]. Special care was taken to determine the children’s age with accuracy from the day care centres’ records. The BMI-for-age, height-for-age and weight-for-height indices expressed in Z-scores were computed using the 1978 National Center for Health Statistics/WHO reference using Epinut software (Epi Info Version 6, Centers for Disease Control) [37].

Feeding practices were assessed through a qualitative 24-h recall, which was reported by the mothers. The food consumed by the toddlers in the day care centres was recorded by the researcher for the 3 days of the test period.

### Determination of sleep patterns

Objective sleep assessment using actigraphy to determine sleep patterns was captured using the Philips Actiwatch 2, (Philips Healthcare, Andover, MD, USA). The Actiwatch device uses an accelerometer to detect and log wrist movement. Actigraphy has been shown to be a useful means for discriminating sleep from wake activity. Actigraphy assesses sleep in a naturalistic environment. Although actigraphy does not provide data on sleep architecture, one advantage of this technique is that it is unobtrusive and allows for data collection over an extended period of time [38]. Actigraphy is a useful diagnostic tool that can be used to estimate sleep parameters with specialised algorithms in computer software programs. It has been well validated for the estimation of night-time sleep parameters across age groups [38]. Actigraphy was chosen over polysomnography as it captures both day and night-time sleep without restricting the child’s freedom of movement. Moreover, it was easy to monitor the sleep during the day time at the child care centres and also at night at home. It was less burdensome on the child care providers as well as the parents. The toddlers seemed to be well adapted to wearing the actigraph watches.

The Actiwatch data indicated children’s sleep and wake states. Unlike parental report measures, actigraphy does not rely on observable behaviour to determine sleep states. An actigraph will accurately record a child’s state as awake when he or she is in a state of quiet wakefulness, whereas his or her parent would be likely to report that the child is asleep. The Actiwatch was worn on the wrist of the child on the day before the administration of the test products to familiarise the child with the watch. The Actiwatch was worn by the child for a period of 3 days. The parents were requested to monitor that the child did not remove the watch when at home. The watch was removed after the study period. The obtained information was analysed using Actiware software version 6.0. Actiware software quantifies important sleep statistics such as sleep time, sleep efficiency and wake after sleep onset based upon user defined or automatically determined rest intervals. These features are designed to simplify analysis of activity data for sleep.

Sleep variables included TST, WASO, SOL and SE. TST represented the total amount of sleep in minutes (computed in hours for the present study) from onset of sleep to onset of awakening. WASO referred to the time (minutes) spent awake after sleep onset had occurred. SOL was the time period measured from “lights out” or “bedtime” to the beginning of sleep, and SE was defined as the proportion of sleep in the period potentially filled by the ratio of total sleep time to time in bed as a percentage, where a SE less than 85 % is considered to be indicative of sleep disturbance [39].

### Statistical analysis

Descriptive statistics were performed to report the data as mean ± S.D. Sociodemographic characteristics and sleep outcomes were compared among both groups using the *t*-test and chi-square test for continuous and categorical data, respectively. All statistical analyses were evaluated at a significance level of 5 % (two-tailed) and were conducted using IBM software version 21.

## Results

### Participants’ profiles

The participants were recruited from July 2012 to April 2013 from 9 day care centres in Klang Valley and Putrajaya. Of the 75 eligible families who agreed to participate in the study, only 56 (74.6 %) consented. Hence, a total of 56 toddlers (37.5 % boys and 62.5 % girls) participated in the study with a mean age of 19.9 ± 4.3 years. About 43.6 % of the toddlers were in the age range of 14–18 months, with 56.4 % of them being in the age range of 19–24 months. The majority of the toddlers were girls (62.5 %) with most (96.4 %) of them belonging to Malay ethnicity. Only 3.6 % of the toddlers belonged to other ethnicities. The majority of the toddlers’ parents (mothers 57.1 %; fathers 37 %) were aged less than 30 years, with a mean age of 30.9 ± 4.1 years for the mothers and 33.3 ± 5.3 years for the fathers (Table 2). Socioeconomic statuses of the families (Table 2) revealed that 34 % of the families had a household monthly income of less than RM 4000 (USD 1333). The majority (58.3 %) of the toddlers had one to two siblings. Most (92.4 %) of the toddlers who participated in the study had a normal birth weight (2500–4000 g) [36].

**Table 2:** Sociodemographic profile of the subjects

Characteristics (*n* = 56)	LGI *n* (%)	HGI *n* (%)	Total *n* (%)
Age (months)			
14–18	11 (39.3)	14 (51.9)	24 (43.6)
19–24	17 (60.7)	13 (48.1)	31 (56.4)
Mean ± S.D.	20.46 ± 3.8	19.30 ± 4.7	19. 9 ± 4.3
Sex			
Male	10 (34.5)	11 (40.7)	21 (37.5)
Female	19 (65.5)	16 (59.3)	35 (62.5)
Ethnicity			
Malay	27 (96.4)	27(96.4)	54 (96.4)
Others	1 (3.6)	1(3.6)	2 (3.6)
Mother’s age (years)*(n=47)*			
≤30	18 (62.1)	14 (51.9)	32 (57.1)
31–35	7 (24.2)	8 (29.6)	15 (26.8)
Mean ± S.D.	30.5 ± 4.1	31.30 ± 4.3	30. 9 ± 4.1
Father’s age (years) (*n* = 54)			
≤30	8 (29.6)	12 (44.5)	20 (37.0)
31–35	12 (44.5)	5 (18.5)	17 (31.5)
>36	7(25.9)	10(37.0)	17 (31.5)
Mean ± S.D.	32.8 ± 4.4	33.8 ± 6.1	33.3 ± 5.3
Mother’s education (*n* = 56)			
Secondary	9 (31.0)	5(18.5)	14(25.0)
College/University	20 (69.0)	22 (81.5)	42 (75.0)
Father’s education (*n* = 54)			
Secondary	7 (25.9)	4(14.8)	11(20.4)
College/University	20 (74.1)	23 (85.2)	43 (79.6)
Mother’s occupation [53] (*n* = 55)			
Managers and professionals	4 (13.8)	10 (38.4)	14 (25.4)
Technicians/Associate professionals	14 (48.3)	6 (23.2)	20 (36.4)
Clerical support workers	10 (34.5)	8 (30.8)	18 (32.7)
Service and sales workers	0 (0.0)	1 (3.8)	1 (1.8)
Business owner	1 (3.4)	1 (3.8)	2 (3.7)
Father’s occupation [53] (*n* = 52)			
Managers and professionals	7 (25.9)	6 (24.0)	13 (25.0)
Technicians and associate professionals	11 (40.8)	13 (52.0)	24 (46.1)
Clerical support workers	2 (7.4)	1 (4.0)	3 (5.8)
Service and sales workers	3 (11.1)	0 (0.0)	3 (5.8)
Plant and machine operators and Assemblers/ Elementary occupations	2 (7.4)	2(8.0)	4 (7.7)
Business owner	2 (7.4)	3 (12.0)	5 (9.6)
Household monthly income (*n* = 56)			
<4000	12(41.4)	7(26.0)	19(34.0)
RM4000 – 5999	7 (24.1)	9 (33.3)	16 (28.6)
RM6000 – 7999	7 (24.1)	7 (25.9)	14 (25.0)
≥RM8000	3 (10.4)	4 (14.8)	7 (12.4)
Number of siblings (*n* = 55)			
No siblings	1 (3.4)	1 (3.8)	2 (3.6)
1–2	15 (51.7)	17 (65.4)	32 (58.3)
3–4	11 (37.9)	8 (30.8)	19 (34.5)
≥5	2 (6.9)	0 (0.0)	2 (3.6)
Child’s Birth Weight (kg) (*n* = 53)			
Low birth weight [37] (<2.5)	1 (3.4)	2 (8.3)	3 (5.7)
Normal birth weight (2.5–3.99)	27 (93.2)	22 (91.7)	49 (92.4)
High birth weight (≥4.00)	1 (3.4)	0 (0.0)	1 (1.9)
Mean ± S.D.	3.1 ± 0.4	3.00 ± 0.5	3.1 ± 0.4

MASCO [53], Principles of Classification of Occupations (3rd Ed.). Putrajaya: Ministry of Human Resources

UNICEF and WHO [36]

### Anthropometric profiles

The BMI for age (Table 3) of the toddlers found 74.5 % being of normal weight, with 12.7 % at risk of overweight, 5.5 % overweight and 7.3 % underweight.

**Table 3:** BMI for age, height for age, weight for age, and weight for height classifications for LGI and HGI groups

	LGI	HGI	Total
	*n* (%)		
BMI for age	*n* = 28	*n* = 27	*n* = 55
Overweight	0 (0.0)	3 (11.1)	3 (5.5)
Risk of overweight	4 (14.3)	3 (11.1)	7 (12.7)
Normal	23 (82.1)	18 (66.7)	41 (74.5)
Under weight	1 (3.6)	3 (11.1)	4 (7.3)
Mean ± S.D. (z-scores)	−0.13 ± 1.06	0.11 ± 1.33	−0.01 ± 1.20
Height for age	*n* = 28	*n* = 27	*n* = 55
Normal	23 (82.1)	20 (74.1)	43 (78.2)
Stunted	4 (14.3)	2 (7.4)	6 (10.9)
Severely stunted	1 (3.6)	3 (11.1)	4 (7.3)
Too tall	0 (0.0)	2 (7.4)	2 (3.6)
Mean ± S.D. (z-scores)	−0.84 ± 1.27	−0.81 ± 2.28	−0.82 ± 1.82
Weight for age	*n* = 28	*n* = 21	*n* = 49
Normal	25 (89.3)	17 (81.0)	0 (0.0)
Underweight	2 (7.1)	2 (9.5)	3 (5.5)
Severely underweight	1 (3.6)	2 (9.5)	7 (12.7)
Mean ± S.D. (z-scores)	−0.61 ± 1.01	−0.46 ± 1.44	−0.54 ± 1.23
Weight for height	*n* = 29	*n* = 27	*n* = 56
Overweight	0 (0.0)	3 (11.1)	3 (5.4)
Risk of overweight	3 (10.3)	1 (3.7)	4 (7.1)
Normal	25 (86.2)	21 (77.8)	46 (82.1)
Underweight	1 (3.5)	2 (7.4)	3 (5.4)
Mean ± S.D. (z-scores)	−0.23 ± 1.03	−0.05 ± 1.23	−0.16 ± 1.13

### Feeding practices during the study period

All of the licensed day care centres followed a recommended menu of the MOH, Malaysia. The meals provided to the toddlers at the day care centres could not be controlled for isocaloric standards. The study did not control for the meals provided at home. The total amount of the test feed consumed by each toddler per day ranged from 540 to 720 mL with 180 mL per feeding.

### Sleep characteristics

The mean TST of the LGI and the HGI groups were 6.91 ± 1.51 and 6.90 ± 1.64 h, respectively (Table 4). The SOL for both groups was 13.27 ± 7.97 (LGI) and 13.07 ± 9.38 (HGI) min. However, there were no significant differences between groups with respect to TST, SOL, WASO and SE.

**Table 4:** Sleep patterns

Test parameters	Measures	HGI (*n* = 28)	LGI (*n* = 28)	*p* value
Total sleep (hours)	Mean ± SD	6.90 ± 1.64	6.91 ± 1.51	0.560
	Median	6.58	7.09	
	Interquartile range	1.22	1.15	
Sleep Onset Latency (SOL)(minutes)	Mean ± SD	13.07 ± 9.38	13.27 ± 7.97	0.724
	Median	13.06	12.2	
	Interquartile range	11.31	14.54	
Wake after sleep onset (WASO) (minutes)	Mean + SD	13.29 ± 10.25	16.41 ± 9.38	0.158
	Median	9.56	17.25	
	Interquartile range	8.94	15.56	
Sleep efficiency (%)	Mean ± SD	86.84 ± 5.89	88.29 ± 5.01	0.442
	Median	87.34	89.05	
	Interquartile range	7.51	6.49	

(Significance level at *p* < 0.05)

## Discussion

Cross-sectional and epidemiologic studies have demonstrated connections between sleep duration and diet. Many studies have indicated that an unhealthy diet is associated with shorter sleep duration and irregular sleeping patterns, despite differences in study methodology [38]. However, these studies have not reported on the quality of the macronutrients and the impact on sleep, especially among toddlers. CHO plays an important role in modulating sleep patterns, which is evident from many studies on adults and school children. However, studies on sleep patterns of toddlers consuming foods with different CHO quality have not been conducted [35].

The present study is possibly the first to test the short-term impact of milk with differing GI amongst toddlers. This pilot study was carried out among 56 toddlers with a mean age of 19.9 ± 4.3 months, and was undertaken to evaluate the short-term impact of LGI and HGI test products on the sleep patterns of toddlers. No significant differences in the sleep patterns of toddlers consuming milk with differing GI values was observed. A possible explanation of this effect could be the short duration of the milk administration. If the study period would have been over a longer duration, the results may possibly have been different. Additionally, the complementary foods consumed by the toddlers were not controlled. Moreover, some of the toddlers were breast fed during the night. Given that the GI of breast milk is similar to that of the LGI test product, the difference of the impact of LGI could not be observed. However, the study demonstrates a slightly better trend in the sleep characteristics among the LGI group as compared with the HGI. Similar results were also reported by a study conducted among eight children (age 8–12 years) of both sexes; the mean SOL, rapid eye movement (REM) latency, TST and TWT were not significantly different in the LGI and HGI groups [40]. A study among toddlers reported that intake of CHO was accompanied by longer sleep duration (0.8 min/g) [35]. However, this study did not examine the GI of the CHO. It was reported that HGI induces an increased sleep duration.

Dietary CHO contain a large variety of sugar chains with different metabolisms. Thus, it is not surprising that the amount of any individual CHO has no consistent influence on sleep parameters. A cross-sectional study on toddlers reported that the consumption of foods with high GI was accompanied by longer sleep time (1.3 min/g) [35]. However, this result seems to be inconsistent with our study, possibly because of the short duration of test feed consumption. Another study among 12 healthy young men with normal weights reported that SOL was significantly different between the LGI and HGI meals given 4 h before bedtime and between the HGI meals given 4 h and 1 h before bedtime [41]. A study of a low-CHO diet on sleep behaviours among six healthy female individuals showed no significant change in sleep time [42]. However, neither a high- nor low-CHO diet affected the sleep parameters.

The toddler and preschool years are generally considered the most difficult phase of life to study because toddler performance is influenced by factors that are outside of experimental control such as emotional state, motivation, persistence and comprehension of instructions. Thus, less research has been done in the toddler years not only because of this age-related variability, but also because there has been a greater emphasis on measures of overall cognitive development such as “IQ”, which is notably difficult to assess until elementary school years [43–45].

A cross-sectional study among toddlers showed that the consumption of foods with a high GI was accompanied by longer sleep time (1.3 min/g) [35, 46]. This may be because macronutrients stimulate the secretion of cholecystokinin, which has been reported to induce sleepiness [35, 44]. However, in a clinical trial of 12 healthy young men consuming CHO meals with high or low GLs, profiling the type of CHO ingested did not affect sleep duration or any other sleep indexes according to polysomnographic recordings, except for the onset of sleep [39]. From the same study, it was observed that shortened SOL by approximately 10 min after a CHO-rich evening meal with a HGI compared with a LGI meal suggested a difference between the CHOs. The CHO content of these rice-based meals was more than 90 % of the total energy content, and the meals were ingested 4 h before bedtime [39].

According to recent studies, HGI CHOs have the ability to increase the ratio of circulating Trp to LNAAs (Trp:LNAA) via a direct action of insulin, which promotes the selective muscle uptake of LNAAs [47]. The mechanism by which an HGI CHO meal shortens SOL is currently unknown. A possible mechanism may be that an HGI meal works through an increased plasma concentration of insulin and Trp to a large neutral amino acid ratio (Trp:LNAA) and its ability to compete for entry into the brain with other LNAAs. The entry of Trp into the brain is linked to its concentration relative to other LNAAs and the main determinant of brain serotonin concentration is a high plasma Trp:LNAA [17]. It is now known that the plasma Trp:LNAA is affected by dietary CHO and protein [21, 46].

In another study by Afaghi *et al.* [39] conducted among adults, a standard isocaloric meal (3212 kJ; 8 % of energy as protein, 1.6 % of energy as fat, and 90.4 % of energy as CHO) was provided to the study subjects with a GL of 81.3 and 175 for the LGI and HGI meals, respectively [39]. This enabled the standardisation of the meals for both the groups. However, in the present study, given that the subjects’ belonged to different day care centres, it was not possible to standardise the meals. Though the day care centres followed the menu instructions provided by the MOH, there could have been differences in the preparation methods and the ingredients mix. The caretakers of the day care centres reported that they would also make suitable changes if they found that the meals provided were not palatable to the toddlers. However, this was also not standardised across the recruited day care centres. Moreover, the food consumed at home was also beyond the control of the researchers. The macronutrient content of the meals may have obscured the impact of the test products.

An HGI meal resulted in a significant (*P* = 0.009) shortening of SOL in healthy sleepers compared with an LGI meal [35]. A study of a low-CHO diet on sleep behaviours in six healthy female individuals showed no significant changes in sleep time [48]. Mindell’s study on cross-cultural differences in infant and toddler sleep reported that predominantly Asian countries had significantly later bed times and shorter TSTs [49]. The timing [46, 49, 50] of the HGI [41] meal also affects sleep onset. An HGI meal ingested 4 h before bedtime was more effective in shortening the SOL than was the same meal ingested 1 h before bedtime [48].

Another study among toddlers reported that higher energy intakes with the evening meal were associated with a longer sleep duration (1 min/10 kcal, *p* < 0.01). With respect to absolute intakes, carbohydrates (0.8 min/g, *p* < 0.0001), especially from HGI foods (1.3 min/g, *p* < 0.01) and a higher GL (1.5 min/g GL, *p* < 0.01) were accompanied by longer sleeping time [49]. A meal consumed close to bedtime is associated with sleep disturbance [46]. Macronutrients [50–52] influence sleep through Trp, which serves as a precursor for brain serotonin, a sleep-inducing agent [49].

Further studies are suggested with a larger population to confirm the trends found in the present study. The non-significant results between the groups may also be attributed to the fact that the test products were administered for a very short time. Some of the toddlers continued breast feeding (at night only), which may also have interfered with the effect of the test products. Given that the GI of breast milk is very low, this may have overshadowed the effects of the test products.

## Conclusions

In the present study, the sleep patterns of toddlers administered milk with a LGI did not differ from those consuming HGI milk formulations. This further strengthens the fact that there is no need to have any added sugars used for the GUM to improve the sleep patterns of this age group. The LGI formulations are preferable as these tend to be higher in dietary fibre and low in refined sugars and starches. Hence, a LGI GUM may be favourably used for this segment of the population, which would also prevent the child from the burden of over nutrition during their later years, thereby potentially reducing the pandemic of obesity. Future studies should consider the glycaemic effects of other foods, along with milk with differing GI, consumed for a longer feeding duration.

Study limitations include the small sample size and a lack of generalizability to other groups. The parents at the day care centres insisted on revealing the product identity. As the present study was double blinded, the protocol did not approve of divulging the product name. This was unacceptable to the parents, who therefore dropped out of the study. Given that mothers in the urban areas are generally well informed, they continued to breast feed their children for longer durations. Thus, excluding the breast-fed toddlers was a bigger challenge. To overcome this challenge, we included the toddlers who were breast fed at night only. Moreover, the complementary foods provided at the day care centres and at home were not standardised. This may have impacted the performance of the test products. However, owing to the growing evidence on the effects of GI on sleep, further studies can be carried out amongst the same age group of non-breast-fed toddlers with standardisation of their complementary foods.

## Abbreviations

Trp: Tryptophan
LNAAs: Large neutral amino acids
GI: Glycaemic index
GL: Glycaemic load
T2DM: Type 2 diabetes mellitus
Moh: Ministry of Health, Malaysia
DHA: Docosahexaenoic acid
GUM: Growing up milk
LGI: Low glycaemic index
HGI: High glycaemic index
WHO: World Health Organization
CHO: Carbohydrates
TST: Total sleep time
SE: Sleep efficiency
WASO: Wake after sleep onset
SOL: Sleep-onset latency
REM: Rapid eye movement
TWT: Total wake time
